# Features associated with the use of single or combined reconstructions: analysis of 1,124 Mohs surgeries^☆^

**DOI:** 10.1016/j.abd.2024.07.009

**Published:** 2025-01-31

**Authors:** Felipe Bochnia Cerci, Letícia Mari Tashima, Stephanie Matthews, Stanislav N. Tolkachjov

**Affiliations:** a Dermatology, Clínica Cepelle, Curitiba, PR, Brazil; b Postgraduate Program - Internal Medicine and Health Sciences, Universidade Federal do Paraná, Curitiba, PR, Brazil; c Dermatology Service, Hospital Universitário Evangélico Mackenzie, Curitiba, PR, Brazil; d School of Medicine, University of Kansas Medical Center, Kansas City, Kansas, United States; e Dermatology, Epiphany Dermatology, Dallas, Texas, United States; f Department of Dermatology, The University of Texas at Southwestern Medical Center, Dallas, Texas, United States; g Division of Dermatology, Baylor University Medical Center, Dallas, Texas, United States; h Texas A&M University College of Medicine, Dallas, TX, United States

Dear Editor,

The idea that a surgical defect should always be completely restored with a single method is not always absolute. In certain cases, combining repair methods after skin cancer removal can be beneficial.^1^ Combined reconstruction can be used in defects that affect two or more facial anatomical units, wounds of extensive dimensions or with limited reservoirs of adjacent tissue, as well as in simpler cases.^2^, ^3^ Although multi-subunit or complex defects are commonly encountered by dermatological surgeons, there are few publications on their frequency, and to our knowledge, no publications about the factors related to their use.^4^, ^5^, ^6^, ^7^, ^8^, ^9^, ^10^ The objective of this study is to assess the frequency of combined reconstructions after Mohs Micrographic Surgery (MMS) and to identify wound and patient characteristics related to their use.

This cross-sectional study includes data from consecutive surgeries performed by the same author (FBC) from April 2018 to March 2023. Information was inserted into the database immediately after each surgery to reduce the risk of bias. The study received IRB approval. The following variables were evaluated: age, sex, diagnosis, anatomical units/subunits involved, wound size and reconstruction method(s). These were classified into primary closure, second intention healing, skin graft, cartilage graft and flaps. This last group, is subdivided into rotation, advancement, transposition, interpolation and hinge flaps. Combination closure was defined as the use of more than one repair method for the same wound.

Inclusion criteria were surgical defects of patients submitted to MMS reconstructed by the same surgeon. Exclusion criteria were wounds reconstructed by other specialties. Data was analyzed using the SPSS™ program (version 22.0, IBM). For statistical analysis, Student's *t*-test and Fisher's exact test were used; the significance of 5% was considered. One thousand one hundred and twenty four surgical defects from 850 patients were included. Thirty-six cases were excluded because they were restored by other specialties. Table 1 describes demographic and surgical data.

**Table 1:** Characteristics analyzed for the use of combined reconstruction.

	Total (n = 1124)	Combined reconstruction (n = 242)	Single reconstruction (n = 882)	p-value
Sex				0.620
Female	638 (57%)	134 (55%)	504 (57%)	
Male	486 (43%)	108 (45%)	378 (43%)	
Age (years)				
<60	335 (32%)	79 (33%)	276 (31%)	0.690
≥60	769 (68%)	163 (67%)	606 (69%)	
**Size**				
≤2 cm	938 (83%)	157 (65%)	781 (89%)	<0.001
>2 cm	186 (17%)	85 (35%)	101 (11%)	
Number of affected anatomical units				<0.001
1	1000 (89%)	164 (68%)	836 (94.8%)	
>1	124 (11%)	78 (32%)	46 (5.2%)	
Specific anatomical subunits				
Ala and nasal sidewall	36 (3.2%)	19 (7.9%)	17 (1.9%)	<0.001
Nasal sidewall and cheek	24 (2.1%)	18 (7.4%)	6 (0.7%)	<0.001
Nasal ala and cheek	22 (2%)	17 (7%)	5 (0.6%)	<0.001
Vermilion and upper cutaneous lip	9 (0.8%)	5 (2%)	4 (0.4%)	0.021

Combined reconstruction was used in 242 cases (21.5%). Fig. 1 illustrates the most frequent combined reconstructions used. Among the combined reconstruction cases, 180 combined two methods, and 62 combined three or more. Defects >20 mm in diameter (35% vs. 11%, p < 0.001; OR = 4.2) and wounds that involved more than one anatomical unit (32% vs. 5.2%, p < 0.001; OR = 8.6) more commonly underwent combined repairs (Table 1).

**Fig. 1:**
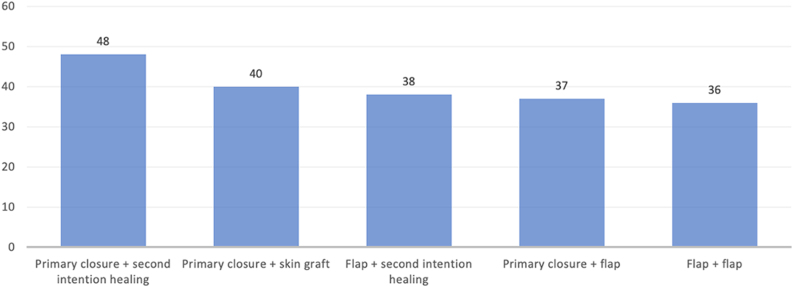
Most frequent combined reconstructions.

Regarding the location, wounds on the nose were more often repaired with a combined method when compared to other facial anatomical units (Table 2). Furthermore, defects involving the following subunits separated by concavity (nasal sidewall/cheek, nasal sidewall/ala, and nasal ala/cheek) or skin/mucosa concomitantly (vermillion/upper cutaneous lip) were more frequently restored with combined methods (Table 1).

**Table 2:** Demographic and surgical data.

	Total (n = 1124)	Combined reconstruction	Single reconstruction
Sex			
Female	490 (58%)		
Male	360 (42%)		
Median age (years)	62.1 (26‒93)		
Fitzpatrick Skin Phototype			
1	64 (7.4%)		
2	439 (52%)		
3	342 (40%)		
4	5 (0.6%)		
Histological subtypes			
BCC	1029 (92%)		
SCC	60 (5%)		
Bowen’s disease	23 (2%)		
Others^a^	12 (1%)		
Combined reconstruction	242 (21.6%)		
Single reconstruction	882 (78.4%)		
Anatomical units^b^			
Nose	468 (41.7%)	130 (27.8%)	338 (72.2%)
Ear	77 (6.9%)	19 (24.7%)	58 (75.3%)
Periocular^c^	61 (5.4%)	11 (18%)	50 (82%)
Perioral	69 (6.1%)	12 (17.4%)	57 (82.6%)
Scalp	46 (4%)	8 (17.4%)	38 (82.6%)
Malar	186 (16.6%)	27 (14.5%)	159 (85.5%)
Forehead/temple^d^	157 (14%)	16 (10.1%)	141 (89.9%)
Others	60 (5.3%)	9 (15%)	51 (85%)

a Others: basosquamous (n = 5), keratoacanthoma (n = 3), dermatofibrosarcoma protuberans (n = 2), atypical fibroxanthoma (n = 1), sebaceous carcinoma (n = 1).

b The main affected anatomical unit of each wound was considered.

c Periocular includes: eyelids, medial and lateral canthus.

d Eyebrow and glabella included in this group.

Although most defects can be adequately restored using a single method, the combined approach has been increasingly reported in the literature and was frequently used in the present study.^8^, ^9^, ^10^ Previous studies described rates of combined reconstruction varying from 7.6% to 13%, lower than the current study.^6^, ^7^

As reported by Patel et al., combined reconstruction is useful in wounds that affect the nasal sidewall and ala simultaneously, as preservation of the alar sulcus is essential for facial symmetry.^8^ Fig. 2 provides a surgical example of a combined reconstruction involving the nasal ala, apical triangle, and upper cutaneous lip. In the present study, wounds involving these subunits were the most frequent site of combined methods. A similar philosophy occurred in defects in the nasal sidewall/cheek, with the objective of recreating the nasofacial sulcus.^10^ This concept was well described in 2004 by Robinson et al. when the author associated repair methods in wounds that affected more than one anatomical unit.^1^

**Fig. 2:**
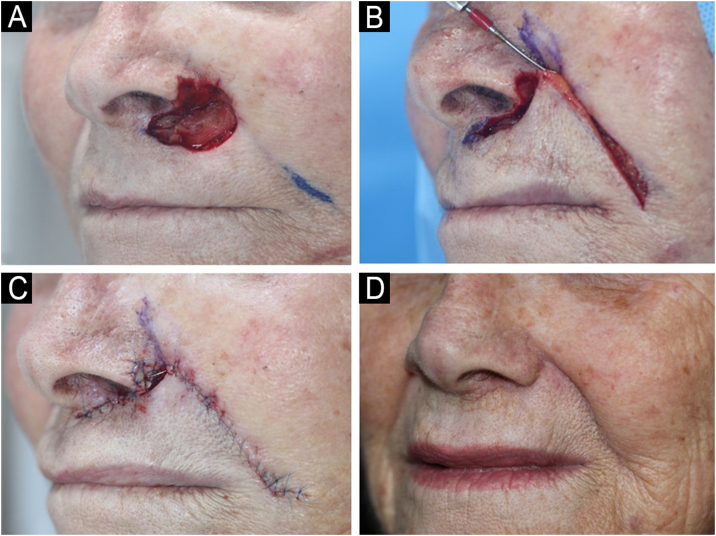
(A) Surgical defect after 3 stages of MMS involving multiple subunits: nasal ala, apical triangle and upper cutaneous lip. (B) Rotation flap movement. (C) Immediate postoperative. Upper cutaneous lip and apical triangle were restored with a nasolabial rotation flap whereas the nasal ala was restored with a full-thickness skin graft. A small area was left to heal by second intention to help recreate the concavity between the nose and the apical triangle. (D) Two months postoperative.

Larger wounds were associated with the combination of methods, which can be explained by the limited skin reservoir in some anatomical units of the face. However, as demonstrated in the present study, small or multi-subunit wounds can also be candidates for combined reconstruction. For example, by associating primary closure with second intention healing, the granulation area and the healing time are reduced. Another common example is associating primary closure with a graft from the adjacent skin (“Burow's graft”), reducing the size of the graft-receiving area.^9^ This practice is especially useful in anticoagulated patients to avoid large undermining areas and increased risk of postoperative bleeding/hematoma.

A strong point of the study is its sample size. The main limitation is the single institution/surgeon experience, inherently seen in most reconstructive studies. While multiple closure methods can change costs in the United States and other countries, the cost did not vary based on methods of reconstruction in this study population.

In conclusion, combined reconstructions were relatively frequent in this sample, mainly in defects over 2 cm and those that affected subunits separated by concavity. Knowledge of this data is relevant, as associating reconstruction methods can be a useful tool for dermatological surgeons when discussing future reconstruction with patients to best tailor expectations.

## Financial support

None declared.

## Authors’ contributions

Felipe Bochnia Cerci: Approval of final version of manuscript, critical literature review, data collection/analysis/interpretation, manuscript critical review, preparation and writing of manuscript, statistical analysis, study conception and planning.

Letícia Mari Tashima: Approval of final version of manuscript, critical literature review, data collection/analysis/interpretation, preparation and writing of manuscript, statistical analysis, study conception and planning.

Stephanie Matthews: Approval of final version of manuscript, manuscript critical review, preparation and writing of manuscript, critical literature review.

Stanislav N. Tolkachjov: Approval of final version of the manuscript, effective participation in research orientation, manuscript critical review.

## Conflicts of interest

None declared.

## References

[1] Robinson J.K. (2004). Segmental reconstruction of the face. Dermatol Surg..

[2] Cerci F.B., Tolkachjov S.N., Cerci F.B., Fantini B.C. (2022). Retalhos e Enxertos em Cirurgia Micrográfica de Mohs.

[3] Scott J.F., Garland K., Bordeaux J.S. (2022). Repair of a defect on the central face involving 9 cosmetic subunits. Dermatol Surg..

[4] Frey M.N., Rossini R.C., Cerci F.B. (2018). Purse string-suture combined with second intention healing for temporal region repair. Surg Cosmet Dermatol..

[5] King B.J., Tolkachjov S.N. (2020). “West by East-West”: combination repair of wide or multiple distal nasal defects. Int J Dermatol.

[6] Nätterdahl C., Kappelin J., Persson B., Lundqvist K., Ahnlide I., Saleh K. (2022). Risk factors for complicated Mohs surgery in the South Sweden Mohs Cohort. J Eur Acad Dermatol Venereol..

[7] Archibald L.K., Gupta R., Shahwan K.T., Swick M., Bakker C., Mattox A.R. (2023). Periorbital reconstructive techniques following Mohs micrographic surgery or excisions: a systematic review. Arch Dermatol Res..

[8] Patel P.M., Greenberg J.N., Kreicher K.L., Burkemper N.M., Bordeaux J.S., Maher I.A. (2018). Combination of melolabial interpolation flap and nasal sidewall and cheek advancement flaps allows for repair of complex compound defects. Dermatologic Surg..

[9] Benoit A., Leach B.C., Cook J. (2017). Applications of Burow’s grafts in the reconstruction of Mohs micrographic surgery defects. Dermatologic Surg..

[10] Tate J.A., Nijhawan R.I. (2023). Repair of a defect involving the nasal sidewall, nasal ala, alar sulcus, and medial cheek. Dermatologic Surg..

